# Multivariate analyses of immune markers reveal increases in plasma EN-RAGE in first-episode psychosis patients

**DOI:** 10.1038/s41398-023-02627-8

**Published:** 2023-10-20

**Authors:** Laura Korhonen, Elisabeth R. Paul, Karin Wåhlén, Liina Haring, Eero Vasar, Antti Vaheri, Dan Lindholm

**Affiliations:** 1 Center for Social and Affective Neuroscience, Department of Biomedical and Clinical Sciences, Linköping University, Linköping, Sweden; 2 Department of Biochemical and Clinical Sciences (BKV), Linköping University, Linköping, Sweden; 3 Department of Child and Adolescent Psychiatry, Linköping University, Linköping, Sweden Region Östergötland, Linköping, Sweden; 4 Pain and Rehabilitation Center, Department of Health, Medicine and Caring Sciences, Linköping University, Linköping, Sweden; 5 Institute of Clinical Medicine University of Tartu; Psychiatry Clinic of Tartu University Hospital, Tartu, Estonia; 6 Institute of Biomedicine and Translational Medicine, University of Tartu, Ravila 19, Tartu, 50411 Estonia; 7 Department of Virology, Medicum, University of Helsinki, 00290 Helsinki, Finland; 8 Department of Biochemistry and Developmental Biology, Medicum, University of Helsinki, PO Box 63, FI-00014 Helsinki, Finland; 9 Minerva Foundation Institute for Medical Research, Biomedicum Helsinki 2U, Tukholmankatu 8, FI-00290 Helsinki, Finland

**Keywords:** Diagnostic markers, Physiology

## Abstract

Immune cells and cytokines are largely recognized as significant factors in the pathophysiology of neuropsychiatric disorders. The possible role of other blood cells such as leukocytes in events of acute psychosis is in contrast only emerging. To study blood-born markers in acute psychosis we here evaluated plasma proteins in drug-naive first-episode psychosis (FEP) patients and healthy controls using a multiplex proximity extension assay technique. We analyzed a panel of 92 immune markers and plasma samples from 60 FEP patients and 50 controls and evaluated the changes obtained using multivariate statistical methods followed by protein pathway analyses. Data showed that 11 proteins are significantly different between FEP patients and healthy controls We observed increases in pro-inflammatory proteins such as interleukin-6, oncostatin-M, and transforming growth factor-alpha in FEP patients compared with controls. Likewise, the extracellular newly identified RAGE-binding protein (EN-RAGE) that regulates the expression of various cytokines was also elevated in the plasma of FEP patients. The results indicate that neutrophil-derived EN-RAGE could play an important role during the early phase of acute psychosis by stimulating cytokines and the immune response targeting thereby likely also the brain vasculature.

## Introduction

Accumulating evidence indicates an association between an altered immune system function and the pathophysiology of psychotic disorders. Pre- and perinatal infections are considered risk factors for the later occurrence of psychotic disorders [1, 2], potentially influencing the immune system and brain development [3, 4]. Genetic studies have delineated immune loci associated with psychosis [5]. An increasing number of studies have also shown changes in inflammatory markers and cytokines in the blood and cerebrospinal fluid of patients with psychotic disorders [6]. However, the profile of immune mediators and inflammatory cytokines (proteins controlling the activity of the immune system) in psychosis patients varies between studies [7–10], making direct comparisons difficult. One reason for this could be confounding factors related to group size, age, smoking, body mass index (BMI) or present medication despite attempts to control these factors [9]. In addition, in the majority of studies, single or small sets of inflammatory proteins have been analyzed. The immune response in humans is a complex and coordinated response involving a multitude of factors and proteins that may have opposite functions that counterbalance each other [11–13]. As previously suggested [6], the assessment of a broad array of proteins involved in the immune response is vital in the search for biomarkers of psychotic disorders.

The aim of this study was to evaluate changes in immune markers in the plasma of drug-naive first-episode psychosis (FEP) patients compared with controls by analyzing 92 proteins related to the immune system using the multiplex proximity extension assay technology developed by Olink. Data obtained were further analyzed using multivariate statistics to reveal alterations in cytokines in antipsychotic-naive FEP patients and in healthy control (HC) subjects. There was an increase in some cytokines such as IL-6 in the FEP patients compared with the controls, which is in line with previous studies [11–13]. We further observed a significant increase in the Extracellular Newly Identified RAGE-binding protein (EN-RAGE) in FEP patients compared with HCs. Increased levels of EN-RAGE have previously been reported in incident coronary heart disease as well as in other inflammation-associated disorders [14–16]. EN-RAGE is expressed by neutrophils and has pro-inflammatory effects by binding to advanced glycosylation end-product receptor (RAGE) that is present on target cells including the vasculature [17]. Our finding of elevated EN-RAGE in acute psychosis is in accordance with recent studies showing enhanced formation of neutrophil extracellular traps (NETs) in the blood of early schizophrenia [18]. We suggest that EN-RAGE and neutrophils may orchestrate the immune response and play an important role during the acute phase of psychosis. The results can also be of help in designing hypotheses for future research about the role of inflammation in acute psychosis.

## Materials and methods

### Study cohorts

This study consisted of 60 first-episode psychosis patients recruited in the Psychiatric Clinic of Tartu University Hospital, Estonia who had no previous contact with the psychiatric care system, and 50 healthy controls who were recruited via advertisement. The study was ongoing between 2009 and 2013. Patients underwent clinical assessment for their eligibility for the study by two psychiatrists according to the International Classification of Diseases, 10^th^ Edition (ICD-10) [19]. FEP patients meet the criteria for a psychotic disorder (F20-F29, see Table 1), they were not on any antipsychotic medication (drug-naive) and had an age between 18 to 45 years. Exclusion criteria for the FEP group were psychotic symptoms induced by some medication or caused by general medical conditions. The onset of the psychotic episode was within the past 3 years at inclusion. HC participants were age-matched. Exclusion criteria for the HC group were any current or previous psychopathology or a family history of psychotic disorders. The general exclusion criteria for all participants were diabetes and neurological or immune-related disorders. A history of substance abuse was not an exclusion criterion as this study was a naturalistic study. Demographic data of the two groups are presented in Table 1. The study was approved by the Ethics Committee, University of Tartu (Dnr 281/T-5). Participants provided written informed consent in accordance with the Declaration of Helsinki. All patient information was anonymized. Some of the samples have been used earlier to study lipid and amino acid alterations in FEP patients [20–22].

**Table 1:** Demographic and clinical data of first-episode psychosis (FEP) and healthy control (HC) groups.

		FEP (*N* = 60)	HC (*N* = 50)		
		Median (IQR)	Median (IQR)	*W*	*p*
Age		26.77 (10.42)	24.90 (7.10)	1751.5	.13
BMI		22.65 (4.63)	22.75 (3.13)	1477	.89
Waist circumference		80.00 (15.13)	77.50 (16.50)	1702	.23
BPRS (*N* = 59)		46.50 (27.00)			
GAF (*N* = 59)		30 (10)			
		%	%	*χ*²	*p*
Sex (female)		46.67	52	0.14	0.71
Alcohol frequency	None	15	16	6.04	0.18
	<once a month	40	60		
	2–4 times a month	33.33	18		
	2–3 times a week	10	6		
	>3 times a week	1.67	0		
Cannabis use	Never	66.67	92	12.04	.005
	Once	5	4		
	A few times	18.33	4		
	Often in the past	3.33	0		
	Regularly	6.67	0		
Smoking	Never	55	76	6.19	.04
	In the past	10	2		
	Current	35	22		
	e-cigarette	0	0		
CGI (*N* = 59)	Moderately ill	10			
	Markedly ill	26.67			
	Severely ill	58.34			
	Among the most extremely ill	3.34			
ICD diagnosis	F20.0 (paranoid schizophrenia)	23.33			
	F20.3 (undifferentiated schizophrenia)	3.33			
	F21 (schizotypal disorder)	1.67			
	F23.0 (acute polymorphic psychotic disorder without symptoms of schizophrenia)	23.33			
	F23.1 (acute polymorphic psychotic disorder with symptoms of schizophrenia)	16.67			
	F23.2 (acute schizophrenia-like psychotic disorder)	25.00			
	F23.3 (other acute predominantly delusional psychotic disorders)	6.67			

*IQR* interquartile range, *BRPS* Brief Psychiatric Rating Scale, *GAF* global assessment of functioning, *CGI* Clinical Global Impression F20.0: paranoid schizophrenia, *F20.3* undifferentiated schizophrenia, *F23.0* acute polymorphic psychotic disorder without symptoms of schizophrenia, *F23.1* acute polymorphic psychotic disorder with symptoms of schizophrenia, *F23.2* acute schizophrenia-like psychotic disorder, *F23.3* acute and transient psychotic disorder.

### Procedures

Overnight fasting blood samples were obtained between 0900 and 1100 h. Venipuncture blood (5 ml) was sampled using VACUETTE heparin tubes, centrifuged for 15 min at 2000 rpm at 4 °C, and the plasma fraction was apportioned into aliquots and stored frozen at –20 °C for up to 2 weeks and then at –80 °C until further analyses. Data about age, sex, body mass index, waist circumference, and smoking, drinking, and cannabis use habits were collected from all participants. The severity of symptoms and sickness were further assessed in FEP participants using the Brief Psychiatric Rating Scale [23], the Global Assessment of Functioning scale [24], as well as the Clinical Global Impression scale [25].

### Inflammatory protein assays

Protein analyses were conducted at two-time points (first run: *N*_FEP_ = 30, *N*_HC_ = 30; second run: *N*_FEP_ = 30, *N*_HC_ = 20). Eight bridging samples were included in the second run to make merging of the two datasets possible. Blood samples were thawed and aliquots were transferred to 96-well plates for subsequent analyses carried out by Olink Proteomics (Uppsala, Sweden) using the Inflammation panel containing in total 92 inflammation related proteins. In this multiplex proximity extension assay (PEA) technology, a pair of oligonucleotides linked to antibodies for each target protein will undergo a real-time polymerase chain reaction, amplifying a sequence that is then quantified. The PEA has a high detection sensitivity in the range of fg/ml protein.

Protein levels were reported as normalized protein expression (NPX) units on a log2-scale. Thus, an increase of 1 NPX represents a two-fold increase of protein level in the sample. As we performed two assays, we conducted a bridge sample normalization to be able to combine data using the “OlinkAnalyze” package in RStudio [26, 27]. Eight samples from the first run were included in the second run as bridge samples. The pairwise differences of the overlapping samples were calculated for each protein. The first run was used as a reference plate, and the median difference was then added to the values of the second run as a plate- and assay-specific normalization factor. For bridging samples, values of the first run were used for the final analyses. The intra-assay CV using the bridging samples varied between 0.82 and 30.77%. Quality controls and pre-processing normalization were conducted by Olink Proteomics. Two samples were flagged during quality control, but no data anomalies were found. Therefore, the samples were included. Values under the limit of detection were kept as the NPX log2 values as provided by Olink. However, proteins with >40 % of values below the limit of detection over all samples were excluded from further analyses, leading to the exclusion of 20 proteins. This gives a number of 72 proteins that were further analyzed in the study. Supplementary Table 1 lists all proteins of the inflammation panel and whether they were included or not.

### Data analysis and statistical methods

#### Demographic and Clinical Data

Using Shapiro–Wilk tests, data were first tested for normality of the clinical and demographic data. Many of the variables were non-normally distributed. We then conducted univariate non-parametric tests (Wilcoxon signed-rank and Chi-square tests) using RStudio [26] to assess differences between FEP and HC groups. Due to a low number of counts in some cells of the Chi-square test, *p*-values were simulated using 2000 replicates for alcohol use frequency, cannabis use, and cigarette smoking. Data are presented as percentages, median, and interquartile ranges. A *p-*value of ≤0.05 was considered statistically significant. In addition, clinical data for the FEP group are presented using percentages, medians, and interquartile ranges.

#### Multivariate data analyses

Multivariate data analyses were performed using SIMCA version 17 (Sartorius Stedim Biotech, Umeå, Sweden). First, a principal component analysis including all proteins and all participants was performed to explore the data. Using distance to model X, the distance between a given observation and the model plane, and Hotellings T2, a multivariate generalization of a 95% confidence interval [27, 28] two strong outliers were detected. We decided to include these participants for further statistical analyses, as no major deviations were found in the individual protein levels.

Next, orthogonal partial least squares regressions discriminant analysis (OPLS-DA) was used to identify a small set of proteins to discriminate between the groups. OPLS-DA is a commonly used method to assess large omics data that model variations in the predictor (in our case the proteins) that are correlated or uncorrelated (orthogonal) to the outcome variable (in our case group belonging).

Mean centering and unit variance scaling were applied to the proteins to assure that low-abundance proteins exert the same influence on the model as high-abundance proteins. The significance of the OPLS-DA model was assessed using cross-validated analyses of variance (CV-ANOVA). Further, goodness of fit and goodness of prediction were assessed using *R*^2^ and *Q*^2^, respectively.

To determine proteins that have the highest discriminant value, we calculated the variable influence in the projection predictive value (VIPpred) statistics, which describe the relative contribution of each protein to the predictive power of the OPLS-DA model. Furthermore, the loadings of each protein on the model plane were scaled as correlation coefficients (*p*(corr)), thereby standardizing the range to –1.0 to 1.0 [29, 30]. A VIPpred ≥1.0 and a *p*(corr) ≥0.3 were used as cut-offs for determining the proteins with the highest discriminant power.

#### Protein pathway analyses

We next examined functional protein–protein association networks between proteins differentially expressed in the FEP and HC samples in the OPLS-DA model. For this, we used the online database tool STRING v11.5, www.string-db.com [31]. Protein accession number (UniProt) of each significant protein and Homo sapiens as the organism were entered into the search engine using multiple proteins. Only query proteins were used as input, and a medium confidence interaction score of 0.40 was used. The input proteins are represented by colored nodes and the protein–protein interaction by a line. The thickness of the line indicates the strength of the interaction.

## Results

Table 1 shows the demographic and clinical data on 60 FEP patients and 50 healthy controls. The FEP and HC groups differed significantly in their cannabis use (*χ*² = 12.04, *p* = 005) and smoking behavior (*χ*² = 6.19, *p* = 04), with the FEP patients having a more frequent history of cannabis or smoking. The majority of FEP patients (71.67%) had an “acute and transient psychotic disorder” (F23) according to the ICD-10 classification. Amongst them, the most common diagnosis was F23.2 (acute schizophrenia-like psychotic disorder, 25.0%), followed by F23.0 (Acute polymorphic psychotic disorder without symptoms of schizophrenia, 23.33%) and F20.0 (paranoid schizophrenia, 23.3%). The majority of patients (61.68%) were considered severely ill on the Clinical Global Impression scale and rated as considerably influenced by their symptoms on the Global Assessment of Functioning scale (median = 30, IQR = 10).

### Levels of immune-related proteins

**Fig. 1:**
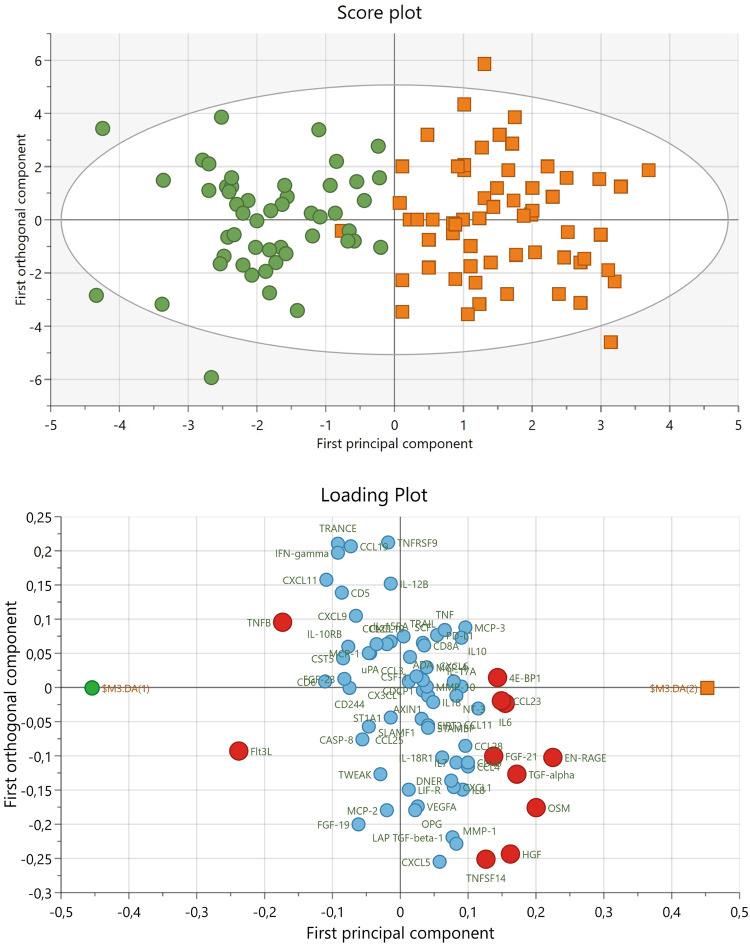
Multivariate data analyses of inflammatory markers in plasma from control and acute psychosis patients. Upper Plot. Score plot of the orthogonal partial least square discriminant analysis (OPLS-DA) model showing a clear separation between first-episode psychosis patients (orange squares) and healthy controls (green circles) along the first principal component. Lower Plot. Loading plot along the first principal component and first orthogonal component. Red dots indicate proteins that significantly differentiate between first-episode psychosis patients (right) and healthy control participants (left) based on variance importance in the projection (VIPpred) value ≥ 1.0 and a *p*(corr) ≥0.3. Significant proteins were: Fms-related tyrosine kinase 3 ligand (Flt3L), protein S100-A12 (EN-RAGE), oncostatin-M (OSM), tumor necrosis factor-beta (TNFB), PRotransforming growth factor-alpha (TGF-alpha), hepatocyte growth factor (HGF), interleukin-6 (IL-6), C–C motif chemokine 23 (CCL23), eukaryotic translation initiation factor 4E-binding protein 1 (4E-BP1), fibroblast growth factor 21 (FGF-21), and tumor necrosis factor ligand superfamily member 14 (TNFSF14). For names of the non-significant proteins see Supplementary Table 1.

We used OPLS-DA data modeling to find the inflammatory proteins that could significantly discriminate between FEP versus HC samples. A significant model with one principal component and two orthogonal components (*F*(6.103) = 15.16, *p* = 2.25e-12) was found, with a high goodness of fit (*R*^2^ = 76), and a moderate goodness of prediction (*Q*^2^ = 47). Figure 1 upper plot shows the separation between the FEP and HC groups. Figure 1 lower plot visualizes proteins that are significantly different between FEP patients and controls. Out of the 72 proteins included, eleven proteins were considered significant discriminators between FEP and HC based on a VIPpred ≥1.0 and a *p*(corr) ≥0.3. These proteins were the Fms-related tyrosine kinase 3 ligand (Flt3L); Protein S100-A12 (EN-RAGE); Oncostatin-M (OSM); Tumor necrosis factor-β (TNFβ); Transforming growth factor-α (TGFα); Hepatocyte growth factor (HGF); Interleukin-6 (IL-6); C–C motif chemokine 23 (CCL23); Eukaryotic translation initiation factor 4E-binding protein 1 (4E-BP1); Fibroblast growth factor 21 (FGF-21); and Tumor necrosis factor ligand superfamily member 14 (TNFSF14). Table 2 depicts VIPpred and *p*(corr) values of proteins that significantly differentiated between FEP patients and HC. Results for all other proteins can be found in Supplementary Table 1.

**Table 2:** Variable influence in the projection (VIPpred) and loadings scaled as correlation coefficients (*p*(corr)) of proteins that significantly differentiate between first-episode psychosis (FEP) and healthy controls (HC).

Uniprot	Protein name	Abbreviation	VIPpred	*p*(corr)	FEP mean (SD)	HC mean(SD)
P49771	Fms-related tyrosine kinase 3 ligand	Flt3L	2.63	–0.6	8.14 (0.42)	8.63 (0.33)
P80511	Protein S100-A12	EN-RAGE	2.49	0.57	3.89 (1)	2.93 (0.78)
P13725	Oncostatin-M	OSM	2.21	0.51	3.33 (1.08)	2.31 (1.02)
P01374	Lymphotoxin-alpha/tumor necrosis factor-beta	TNFB	1.91	–0.44	4.11 (0.66)	4.61 (0.4)
P01135	Protransforming growth factor alpha	TGF-alpha	1.91	0.44	2.94 (0.41)	2.7 (0.22)
P14210	Hepatocyte growth factor	HGF	1.8	0.41	7.44 (0.38)	7.21 (0.25)
P05231	Interleukin-6	IL-6	1.72	0.39	2.5 (1.01)	1.92 (0.53)
P55773	C–C motif chemokine 23	CCL23	1.64	0.38	9.97 (0.49)	9.66 (0.53)
Q13541	Eukaryotic translation initiation factor 4E-binding protein 1	4E-BP1	1.59	0.37	6.58 (0.91)	5.95 (1.19)
Q9NSA1	Fibroblast growth factor 21	FGF-21	1.52	0.35	5.07 (1.24)	4.16 (1.34)
O43557	Tumor necrosis factor ligand superfamily member 14	TNFSF14	1.4	0.32	3.74 (0.54)	3.49 (0.52)

A VIPpred-value ≥ 1.0 and a *p*(corr) ≥ 0.30 was considered significant.

### Protein pathway analysis

**Fig. 2:**
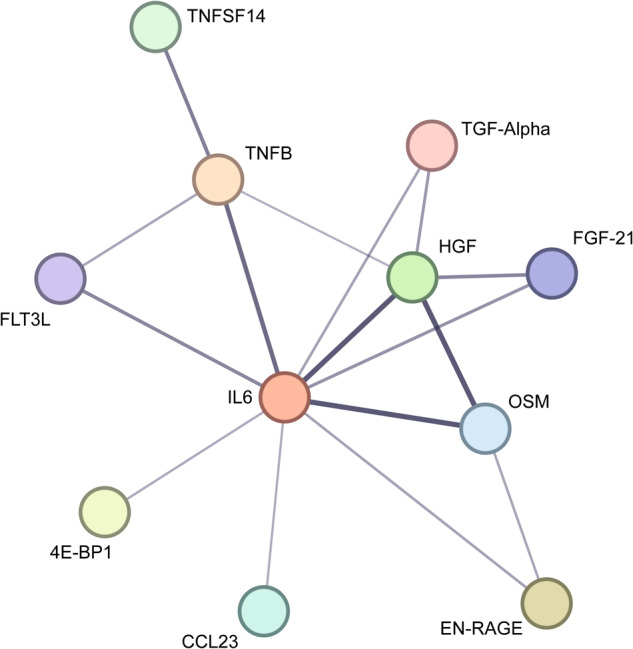
Protein–protein interaction network in acute psychosis. The interaction network was created using the online database tool STRING v 11.5. Proteins depicted are the proteins that significantly differentiated between first-episode psychosis and healthy controls: Fms-related tyrosine kinase 3 ligand (Flt3L), protein S100-A12 (EN-RAGE), oncostatin-M (OSM), tumor necrosis factor-beta (TNFB), protransforming growth factor alpha (TGF-alpha), hepatocyte growth factor (HGF), interleukin-6 (IL-6), C–C motif chemokine 23 (CCL23), eukaryotic translation initiation factor 4E-binding protein 1 (4E-BP1), fibroblast growth factor 21 (FGF-21), and tumor necrosis factor ligand superfamily member 14 (TNFSF14).

We next performed a protein network analysis with regard to the proteins that significantly differentiated between FEP and HC groups. The network of interactions centered around IL-6 (protein–protein interaction enrichment *p* = 1.94e-10, average local clustering coefficient: 0.78) and is shown in Fig. 2. As expected since all proteins are related to immune system functioning, the analyses indicate an interaction between the concerned proteins, with EN-RAGE and OSM being able to stimulate the production of IL-6. CCL23 is a chemokine with known activity for neutrophils and monocytes, whilst TNFβ/Lymphotoxin-α and TNFSF14 are cytokines that are generally increased under stress conditions, and have effects among others on inflammation.

## Discussion

In the present work, we have employed the sensitive multiplex PEA technology to study the pattern of plasma protein alterations occurring during acute psychosis in first-episode drug-naive patients. The groups did not differ with regard to age, gender, or BMI. Results revealed significant changes in 11 distinct proteins of 72 analyzed in plasma, of which 9 were increased and 2 were decreased, in 60 FEP patients compared with 50 age-matched control.

Several of the elevated plasma proteins detected have functional links to the acute phase response that accompanies immune activation, inflammation, and body responses to trauma. IL-6 is a cytokine that plays a key role in the immune response and in stimulating the differentiation of monocytes, lymphocytes, and other cells. The levels of IL-6 are under dynamic regulation by other cytokines and the highest IL-6 levels are usually observed during the acute phase of a tissue reaction [11–13].

OSM is a cytokine that enhances production of IL-6 and it was also increased in the FEP patients compared with controls. Other proteins that were elevated in FEP included TGFα and TNFSF14 both having pro-inflammatory effects, as well as the chemokine CCL23 that influences chemotaxis of blood cells including neutrophils [32]. The growth factor HGF was also increased in FEP plasma and is known to regulate the growth and motility of a variety of cells. Recently it was reported that HGF is elevated in a model of traumatic brain injury, and thus contributes to neuroinflammation [33]. FGF-21 is an endocrine factor important for the regulation of cell and tissue metabolism [34] and it was also increased in the FEP patients. FGF-21 was also shown to influence brain neurons and can afford neuroprotection against different insults [35, 36]. TNFβ/Lymphotoxin-α is a cytokine expressed by lymphocytes and it was decreased in the FEP samples, as was the protein Flt3L. TNFβ was recently shown to be increased in a subgroup of high-risk individuals for schizophrenia in a Chinese population using an enzyme-linked immunosorbent assay [37]. Future studies with larger patient groups and of different ethnicities are required to support the role of TNFβ in acute and chronic schizophrenia patients. Flt3L is important in the regulation of dendritic cells and for the proliferation of early hematopoietic cells, but its role in brain diseases is largely unknown. It was recently reported that Flt3L is increased in micro-dialysis samples of the human brain after traumatic brain injury [38].

Comparing the present data on cytokines with previous studies shows similarities but also some dissimilarities. Thus, the results of several studies show increases in cytokines, such as IL-6 and TNF-alpha, during the acute phase of schizophrenia spectrum disorders as recently reviewed in a meta-analysis report by Goldsmith et al. [39]. We previously observed higher levels of IL-6 in the serum of FEP patients compared with control patients using a high-sensitive biochip array technology [22]. In that study, the levels of IL-2 and of the anti-inflammatory cytokine IL-4 were increased in the serum of FEP patients compared to controls [22]. In the present study, more than 62% of plasma values for IL-4 were missing, why this cytokine was excluded from further analyses. Likewise, the values for IL-2 were not detected in most of the samples so these were also excluded (see Supplementary Table 1). In our previous study, serum samples were employed for the determination of cytokines by using a biochip array method [22], whereas in the current study, we analyzed plasma samples by using the Olink technology. A recent study on schizophrenia using the Olink inflammatory panel and patient samples from plasma and cerebrospinal fluid identified immune blood markers linked mainly to the WNT/β-catenin signaling [40]. There was also an increase in plasma EN-RAGE in the FEP patients which is in keeping with our study. Further investigations using additional cohorts will help to resolve the importance of changes in different cytokines in schizophrenia and in specific patient subgroups.

In the present work, we observed that the factor, EN-RAGE was increased in the plasma of acute psychosis patients compared with controls. The association of EN-RAGE with schizophrenia was also observed previously by using proteomics of salivary proteins. Thus, it was reported that the level of EN-RAGE was increased more than 10-fold in salivary secretion in schizophrenia patients compared to normal controls [41]. This finding is in line with the current study showing an increase in EN-RAGE in the plasma of FEP patients compared with controls.

EN-RAGE is a member of the S100 protein family having two EF calcium-binding motifs [42, 43]. EN-RAGE is expressed mainly by granulocytes and some monocytes in the blood and has been linked to inflammatory diseases as well as to certain tumors [15, 16]. EN-RAGE binds to RAGE (receptor for advanced glycation end products), which is a multi-ligand receptor belonging to the immunoglobulin superfamily of transmembrane proteins. RAGE binds many ligands apart from EN-RAGE, such as AGEs (advanced glycation end products), HMGB1 (high-mobility group box-1), glycosaminoglycans, and amyloid β peptides [17, 44]. There is also a splice variant of RAGE receptor, sRAGE, present in serum and that may act as a decoy receptor by binding EN-RAGE and the other ligands [45]. EN-RAGE/RAGE signaling can enhance the expression of adhesion molecules, such as the intracellular adhesion molecule (ICAM1) and vascular cell adhesion molecule-1 (VCAM1) that are present in the vasculature [17, 44]. EN-RAGE may therefore increase cell adhesion in brain vasculature that could be of importance also during the acute phase of neuropsychiatry diseases. Further studies on EN-RAGE and its receptor RAGE in conjunction with other ligands are warranted in order to fully understand the role of this protein in schizophrenia and other mental disorders.

Recently, it was observed that serum levels of EN-RAGE are increased in patients with incident coronary heart disease, representing an important biomarker for the disease [14]. The increase in EN-RAGE as observed here in the plasma of FEP patients may offer an explanation for the elevated cardiovascular risk found in schizophrenia and other severe mental disorders [46]. However, further studies using replication cohorts and longitudinal markers would be important in order to substantiate the possible role of EN-RAGE for cardiovascular risk in mental disorders.

Recently it was observed that EN-RAGE is elevated in the cerebrospinal fluid in patients infected with SARS-CoV-2 virus [47]. In this work, we show that elevated plasma EN-RAGE is associated with a neuropsychiatric disease as shown here for patients with acute psychosis. We noted that the observed increase in EN-RAGE was a more important discriminator between FEP and HCs, than other cytokines, including IL-6.

Previously, higher amounts of white blood cells have been observed in patients with early psychosis independent of other factors such as age, gender, BMI, or smoking [48]. Recently this issue was studied further by examining the degree of formation of neutrophil extracellular traps (NETs) that are present in various other inflammatory disorders [49]. Results showed an increase in NETs in schizophrenia patients compared with controls, indicating an involvement of neutrophils in the disease process [18]. This is in line with our present findings of higher EN-RAGE levels in acute psychosis as this protein is mainly produced by neutrophils in humans. We, therefore, suggest that the increased levels of EN-RAGE observed may reflect the activation of neutrophils and a subsequent inflammatory response present during the acute phase of psychosis.

In conclusion, our study shows increases in pro-inflammatory cytokines and EN-RAGE levels in blood samples of FEP patients compared with controls. EN-RAGE expressed by neutrophils could constitute a novel, easily accessible, blood-born marker for acute psychosis. There are limitations of this study, such as the relatively low number of patient samples analyzed in both the HC and FEP groups. In addition, although some confounding factors such as age, smoking, and gender are controlled for, other variables such as the patients´ overall status and some unknown factors could possibly also influence the results. It is important to perform additional studies on EN-RAGE and schizophrenia in the future in order to replicate the findings using other cohorts and to evaluate whether EN-RAGE can be a potential longitudinal biomarker for the disease.

Likewise, more biological studies on the roles of RAGE and other ligands binding RAGE in neuropsychiatric disorders and disease models are also warranted.

### Supplementary information

Supplementary Table 1
