# Supplementary material for: Multivariate analyses of immune markers reveal increases in plasma EN-RAGE in first-episode psychosis patients
Source: Transl Psychiatry. 2023 Oct 20;13:326. doi: 10.1038/s41398-023-02627-8 (PMC10589203; doi:10.1038/s41398-023-02627-8)
Supplement: Supplementary file 1 — Supplementary Table 1 [file 41398_2023_2627_MOESM1_ESM.pdf]

Supplementary Table 1. List of all proteins from the inflammatory panel (Olink Proteomics, Uppsala, Sweden) with protein name, and statistics (VIPpred, p(corr), mean and standard deviation of protein levels in first episode psychosis (FEP) patients and healthy controls (HC)

Uniprot	Protein name	Statistics			
		VIPpred	p(corr)	FEP Mean (SD)	HC Mean (SD)
P00813	Adenosine deaminase	0,37	0,09	3.13 (0.82)	3.11 (0.53)
Q5T4W7	Artemin				
O15169	Axin-1	0,45	0,1	3.05 (0.65)	3.03 (0.73)
P01138	Beta-nerve growth factor				
Q14790	Caspase-8	0,61	-0,14	4.3 (0.53)	4.52 (0.82)
Q99616	C-C motif chemokine 13	0,44	0,1	15.1 (0.73)	15.06 (0.7)
Q99731	C-C motif chemokine 19	0,81	-0,19	8.38 (0.69)	8.59 (0.69)
P13500	C-C motif chemokine 2	0,51	-0,12	11.41 (0.52)	11.54 (0.31)
P78556	C-C motif chemokine 20	0,39	-0,09	5.9 (0.65)	6.02 (0.63)
P55773	C-C motif chemokine 23	1,64	0,38	9.97 (0.49)	9.66 (0.53)
O15444	C-C motif chemokine 25	0,52	-0,12	5.37 (0.58)	5.49 (0.49)
Q9NRJ3	C-C motif chemokine 28	1,07	0,25	0.81 (0.32)	0.68 (0.34)
P10147	C-C motif chemokine 3	0,16	0,04	4.04 (0.47)	4.08 (0.42)
P13236	C-C motif chemokine 4	1,09	0,25	5.48 (0.45)	5.31 (0.45)
P80098	C-C motif chemokine 7	1,06	0,24	3.03 (0.7)	2.85 (0.44)
P80075	C-C motif chemokine 8	0,23	-0,05	8.12 (0.67)	8.21 (0.62)
Q9H5V8	CUB domain-containing protein 1	0,37	0,08	1.95 (0.49)	1.89 (0.37)
P02778	C-X-C motif chemokine 10	0,23	-0,05	8.81 (0.96)	8.95 (0.55)
O14625	C-X-C motif chemokine 11	1,2	-0,28	10.82 (0.84)	11.19 (0.39)
P42830	C-X-C motif chemokine 5	0,63	0,15	11.11 (0.85)	10.96 (0.73)
P80162	C-X-C motif chemokine 6	0,87	0,2	10.04 (0.43)	9.9 (0.51)
Q07325	C-X-C motif chemokine 9	0,73	-0,17	6.14 (0.71)	6.39 (0.52)

P28325	Cystatin-D	0,93	-0,21	4.14 (0.46)	4.29 (0.39)
Q8NFT8	Delta and Notch-like epidermal growth factor-related receptor	0,84	0,19	8.7 (0.19)	8.62 (0.23)
P51671	Eotaxin	1,26	0,29	7.31 (0.24)	7.19 (0.25)
Q13541	Eukaryotic translation initiation factor 4E-binding protein 1	1,59	0,37	6.58 (0.91)	5.95 (1.19)
O95750	Fibroblast growth factor 19	0,69	-0,16	7.28 (0.74)	7.44 (1.02)
Q9NSA1	Fibroblast growth factor 21	1,52	0,35	5.07 (1.24)	4.16 (1.34)
Q9GZV9	Fibroblast growth factor 23	0,9	-0,21	1.87 (0.47)	2.08 (0.92)
P12034	Fibroblast growth factor 5				
P49771	Fms-related tyrosine kinase 3 ligand	2,63	-0,6	8.14 (0.42)	8.63 (0.33)
P78423	Fractalkine	0,13	0,03	5.13 (0.34)	5.11 (0.43)
P39905	Glial cell line-derived neurotrophic factor				
P09341	Growth-regulated alpha protein	0,87	0,2	8.79 (0.48)	8.66 (0.46)
P14210	Hepatocyte growth factor	1,8	0,41	7.44 (0.38)	7.21 (0.25)
P01579	Interferon gamma	1,01	-0,23	5.58 (1.08)	6.08 (0.81)
P01583	Interleukin-1 alpha				
P22301	Interleukin-10	1	0,23	2.84 (0.63)	2.65 (0.53)
Q13651	Interleukin-10 receptor subunit alpha				
Q08334	Interleukin-10 receptor subunit beta	0,85	-0,19	5.33 (0.32)	5.46 (0.26)
P29460	Interleukin-12 subunit beta	0,16	-0,04	4.51 (0.79)	4.58 (0.48)
P35225	Interleukin-13				
Q13261	Interleukin-15 receptor subunit alpha	0,16	-0,04	0.54 (0.34)	0.59 (0.26)
Q16552	Interleukin-17A	1	0,23	1.23 (0.5)	1.1 (0.44)
Q9P0M4	Interleukin-17C				
Q14116	Interleukin-18	0,53	0,12	7.56 (0.49)	7.5 (0.48)
Q13478	Interleukin-18 receptor 1	0,69	0,16	7.73 (0.34)	7.66 (0.37)
P60568	Interleukin-2				
P14784	Interleukin-2 receptor subunit beta				
Q9NYY1	Interleukin-20				
Q9UHF4	Interleukin-20 receptor subunit alpha				
Q8N6P7	Interleukin-22 receptor subunit alpha-1				
Q13007	Interleukin-24				
O95760	Interleukin-33				
P05112	Interleukin-4				

P05113	Interleukin-5				
P05231	Interleukin-6	1,72	0,39	2.5 (1.01)	1.92 (0.53)
P13232	Interleukin-7	0,91	0,21	2.09 (0.66)	1.92 (0.51)
P10145	Interleukin-8	1,01	0,23	4.78 (0.52)	4.62 (0.45)
P03956	Interstitial collagenase	0,91	0,21	7.64 (0.89)	7.37 (1.01)
P21583	Kit ligand	0,36	0,08	9.29 (0.28)	9.28 (0.36)
P15018	Leukemia inhibitory factor				
P42702	Leukemia inhibitory factor receptor	0,14	0,03	3.62 (0.24)	3.62 (0.23)
P01374	Lymphotoxin-alpha	1,91	-0,44	4.11 (0.66)	4.61 (0.4)
P09603	Macrophage colony-stimulating factor 1	0,27	0,06	9.12 (0.24)	9.13 (0.19)
Q8IXJ6	NAD-dependent protein deacetylase sirtuin-2	0,46	0,1	4.31 (0.87)	4.27 (0.94)
Q9BZW8	Natural killer cell receptor 2B4	0,84	-0,19	6.46 (0.27)	6.59 (0.27)
P20783	Neurotrophin-3	0,92	0,21	1.5 (0.57)	1.34 (0.45)
Q99748	Neurturin				
P13725	Oncostatin-M	2,21	0,51	3.33 (1.08)	2.31 (1.02)
Q9NZQ7	Programmed cell death 1 ligand 1	0,59	0,14	5.93 (0.35)	5.91 (0.27)
P80511	Protein S100-A12	2,49	0,57	3.89 (1)	2.93 (0.78)
P01135	Protransforming growth factor alpha (TGF-alpha)	1,91	0,44	2.94 (0.41)	2.7 (0.22)
Q13291	Signaling lymphocytic activation molecule	0,35	0,08	0.63 (0.38)	0.61 (0.29)
O95630	STAM-binding protein	0,46	0,11	4.89 (0.7)	4.84 (0.75)
P09238	Stromelysin-2	0,44	0,1	7.8 (0.94)	7.68 (0.59)
P50225	Sulfotransferase 1A1	0,17	-0,04	4.26 (0.9)	4.41 (0.98)
P30203	T-cell differentiation antigen CD6	1,23	-0,28	4.73 (0.43)	4.96 (0.37)
P06127	T-cell surface glycoprotein CD5	0,95	-0,22	4.52 (0.35)	4.67 (0.24)
P01732	T-cell surface glycoprotein CD8 alpha chain	0,39	0,09	9.56 (0.61)	9.48 (0.55)
Q969D9	Thymic stromal lymphopoietin				
P01137	Transforming growth factor beta-1 proprotein(TGF-beta-1)	0,86	0,2	6.87 (0.34)	6.77 (0.38)
P01375	Tumor necrosis factor	0,73	0,17	2.31 (0.87)	2.2 (0.67)
P50591	Tumor necrosis factor ligand superfamily member 10	0,06	0,01	7.06 (0.41)	7.09 (0.31)

O14788	Tumor necrosis factor ligand superfamily member 11	1,01	-0,23	4.29 (0.87)	4.72 (0.64)
O43508	Tumor necrosis factor ligand superfamily member 12	0,32	-0,07	8.94 (0.31)	9.01 (0.37)
O43557	Tumor necrosis factor ligand superfamily member 14	1,4	0,32	3.74 (0.54)	3.49 (0.52)
O00300	Tumor necrosis factor receptor superfamily member 11B	0,25	0,06	9.15 (0.28)	9.1 (0.29)
P25942	Tumor necrosis factor receptor superfamily member 5	1,1	0,25	10.74 (0.3)	10.66 (0.29)
Q07011	Tumor necrosis factor receptor superfamily member 9	0,2	-0,04	6.24 (0.53)	6.31 (0.3)
P00749	Urokinase-type plasminogen activator	0,48	-0,11	9.7 (0.31)	9.8 (0.28)
P15692	Vascular endothelial growth factor A	0,28	0,06	9.04 (0.34)	9.03 (0.33)
